# Antimicrobial prophylaxis is considered sufficient to preserve an acceptable surgical site infection rate in clean orthopaedic and neurosurgeries in dogs

**DOI:** 10.1186/s13028-020-00545-z

**Published:** 2020-09-17

**Authors:** Kirsi Johanna Välkki, Katariina Hanne Thomson, Thomas Sven Christer Grönthal, Jouni Juho Tapio Junnila, Merja Hilma Johanna Rantala, Outi Maria Laitinen-Vapaavuori, Sari Helena Mölsä

**Affiliations:** 1 Department of Equine and Small Animal Medicine, Faculty of Veterinary Medicine, University of Helsinki, P.O. Box 57, Viikintie 49, 00014 Helsinki, Finland; 2 Small Animal Hospital, Faculty of Veterinary Medicine, University of Helsinki, P.O. Box 57, Viikintie 49, 00014 Helsinki, Finland; 3 Oy 4Pharma Ltd, Arkadiankatu 7, 00100 Helsinki, Finland

**Keywords:** Antibiotic, Canine, Prophylaxis, Surgery

## Abstract

**Background:**

Surgical site infections (SSI) are associated with increased morbidity and mortality. To lower the incidence of SSI, antimicrobial prophylaxis is given 30–60 min before certain types of surgeries in both human and veterinary patients. However, due to the increasing concern of antimicrobial resistance, the benefit of antimicrobial prophylaxis in clean orthopaedic and neurosurgeries warrants investigation. The aims of this retrospective cross-sectional study were to review the rate of SSI and evaluate the compliance with antimicrobial guidelines in dogs at a veterinary teaching hospital in 2012–2016. In addition, possible risk factors for SSI were assessed.

**Results:**

Nearly all dogs (377/406; 92.9%) received antimicrobial prophylaxis. Twenty-nine dogs (7.1%) did not receive any antimicrobials and only four (1.1%) received postoperative antimicrobials. The compliance with in-house and national protocols was excellent regarding the choice of prophylactic antimicrobial (cefazolin), but there was room for improvement in the timing of prophylaxis administration. Follow-up data was available for 89.4% (363/406) of the dogs. Mean follow-up time was 464 days (range: 3–2600 days). The overall SSI rate was 6.3%: in orthopaedic surgeries it was 6.7%, and in neurosurgeries it was 4.2%. The lowest SSI rates (0%) were seen in extracapsular repair of cranial cruciate ligament rupture, ulnar ostectomy, femoral head and neck excision, arthrotomy and coxofemoral luxation repair. The highest SSI rate (25.0%) was seen in arthrodesis. Omission of antimicrobials did not increase the risk for SSI (P = 0.56; OR 1.7; CI_95%_ 0.4–5.0). Several risk factors for SSI were identified, including methicillin-resistant *Staphylococcus pseudintermedius* carriage (P = 0.02; OR 9.0; CI_95%_ 1.4–57.9) and higher body temperature (P = 0.03; OR 1.69; CI_95%_ 1.0–2.7; mean difference + 0.4 °C compared to dogs without SSI).

**Conclusions:**

Antimicrobial prophylaxis without postoperative antimicrobials is sufficient to maintain the overall rate of SSI at a level similar to published data in canine clean orthopedic and neurosurgeries.

## Background

Surgical site infection (SSI) is defined as an infection that emerges after surgery [1]. This inherent risk in surgical procedures is associated with increased morbidity, costs, and even death [2–4]. Antimicrobial prophylaxis in surgery refers to antimicrobial administration in the immediate proximity of the surgical procedure. According to the European Center for Disease Prevention and Control, antimicrobial prophylaxis beyond 24 h post-surgery in humans is not recommended in clean and clean-contaminated surgeries. Moreover, the Swedish Council on Health and Technology Assessment states that for closed fractures and elective prosthetic surgery, there is scientific support for limiting antimicrobial administration to prophylaxis only [5]. According to the most recent Centers for Disease Control and Prevention (CDC) guideline for SSI prevention, the recommendation is to administer the surgical prophylactic antimicrobial in time to allow the chosen drug to reach sufficient tissue concentrations at the surgical site before the incision is made [1]. Pre- or postoperative antimicrobial administration is not suggested unless given for other reasons such as treatment of a concurrent infection [1, 6].

In veterinary medicine, the current Nordic recommendations are for reduced use of antimicrobial prophylaxis, and emphasize strict surgical asepsis. According to the Danish Antibiotic Use Guidelines for Companion Animal Practice, prophylaxis is not required for patients with American Society of Anesthesiologists (ASA) classification 1–2 with clean procedures, or for apyretic ASA 3 patients undergoing a clean or clean-contaminated procedure [7]. The respective Swedish guidelines state that antimicrobial prophylaxis is not suggested in clean orthopaedic procedures in low-risk patients, even when a surgical implant other than a joint prosthesis is placed [8]. Finnish guidelines similarly advise that antimicrobial prophylaxis is not recommended in clean and clean-contaminated soft tissue surgeries that take less than 60 min. However, in clean orthopaedic surgeries the recommended protocol consists of a first-generation cephalosporin given 30–60 min before the surgical incision, and the antimicrobial administration is continued until the end of surgery [9]. Our hospital guidelines are in accordance with the Finnish guidelines.

Research results regarding the optimal use of antimicrobials in preventing surgical site infections is inconclusive. Many studies suggest that postoperative antimicrobial administration after clean orthopaedic surgeries is protective against SSI in dogs [10–15]. However, there is also evidence that antimicrobial administration in the postoperative period has no protective effect on the incidence of SSI [16, 17]. Furthermore, recent evidence suggests that the SSI rate in certain neurosurgeries is very low, even without the use of prophylactic antimicrobials [18]. Therefore, stronger evidence either for or against the use of antimicrobial prophylaxis in clean surgeries is required, particularly in light of the alarmingly increasing antimicrobial resistance [2].

Reported SSI rates in canine clean orthopaedic and neurosurgeries range from 0.6% to 7.1% [2, 18, 19]. However, higher SSI rates of up to 21.3% and 25.9% have been reported in tibial plateau levelling osteotomy (TPLO) surgeries [15, 17]. In human medicine, risk factors for surgical site infections include obesity, concurrent diseases, breaks in aseptic technique, prolonged hospitalization and methicillin-resistant *Staphylococcus aureus* (MRSA) colonization [2]. In veterinary medicine, additional risk factors associated with an increased SSI incidence include more people present in the operating room, prolonged duration of anaesthesia and surgery, presence of a drain, concurrent endocrinopathy and the use of propofol [2, 3].

This study aimed to retrospectively review the SSI rate in canine clean orthopaedic and neurosurgeries at the Veterinary Teaching Hospital of the University of Helsinki (Finland) and to relate the SSI rate and perioperative antimicrobial use to previously published data. Further aims were to evaluate compliance with in-house and national antimicrobial guidelines at a veterinary teaching hospital, and to identify possible risk factors for SSIs in order to raise hypotheses for prospective studies. We expected that the SSI rate would be within the previously cited range (0.6–7.1%) and that the compliance with antimicrobial guidelines would be good.

## Methods

### Study design

This was a cross-sectional retrospective study. The study population included dogs that had undergone a clean orthopaedic or neurosurgery between January 2012 and December 2016 at the Veterinary Teaching Hospital of the University of Helsinki. Surgeries comprised arthrodesis, arthroscopy, arthrotomy, cruciate ligament rupture repair, femoral head and neck excision, hemilaminectomy and laminectomy, patellar luxation repair, surgical fracture stabilization, and ulnectomy. Prosthetic surgeries were excluded because this type of surgery was not performed during the study period.

### Data collection

Data were collected from the electronic medical records (Provet Net, FNS Finland) of all dogs that had undergone the aforementioned surgical procedures. Information retrieved included signalment, concurrent diseases, ASA classification, being a risk patient (i.e. screened for the carriage of multidrug resistant (MDR) bacteria; for further information on risk patient classification, see Additional file 1), known carriage of MDR bacteria, administration of antimicrobials (preoperatively: before admission to hospital, prophylactic: parenteral administration of antimicrobials in the immediate proximity to surgery, or postoperatively: beyond 24 h after surgery), timing of antimicrobial prophylaxis in relation to skin incision, administration of local anaesthetic (regional or epidural anaesthesia), duration of anaesthesia (from induction to extubation) and surgery (from skin incision to closure), lowest rectal body temperature and lowest mean arterial blood pressure (MAP) value (when < 60 mmHg), as well as duration of hypotension (MAP < 60 mmHg) during the anaesthesia, whether or not an implant was placed, duration of hospitalization, follow-up visits, bacterial culture results and treatment of SSI. Definitions of SSI followed the CDC guidelines (Table 1) [20]. If signs of SSI, such as redness, pain, swelling, incisional drainage or incisional dehiscence were mentioned, or if a bacterial culture of the surgical site was performed, the dog was considered as having a surgical site infection. If no abnormalities suggesting an SSI were mentioned in the patient record from the post-surgical follow-up visit, the surgical site was considered as not infected.

**Table 1:** Surgical site infection definitions according to the CDC guidelines [20]

Superficial incisional SSI	Infection occurs within 30 days of surgery AND Involves only skin or subcutaneous tissue of the incision AND At least ONE of the following: Purulent drainage Positive microbial culture from the incision site At least one of the following symptoms: pain or tenderness, localized swelling, redness or heat Diagnosis of SSI is made by a veterinarian
Deep incisional SSI	Infection occurs within 30 days of surgery or within 1 year if implant is in place AND Involves deep soft tissue (fascia, muscle etc.) of the incision AND At least ONE of the following: Purulent drainage from the deep incision Deep incision dehisces or is opened by the surgeon when the dog has at least one of the following symptoms UNLESS bacterial culture of the incision is negative: Fever Localized pain or tenderness An abscess or other evidence of infection in the deep soft tissues of the incision is found on examination, reoperation, histopathology or imaging
Organ/Space SSI	Infection occurs within 30 days of surgery or within 1 year if implant is in place AND Involves any area other than the incision which was opened or manipulated during surgery AND At least ONE of the following: Purulent drainage Positive bacterial culture An abscess or other evidence of infection involving the organ/space found on examination or by histopathology or diagnostic imaging Diagnosis of SSI made by a veterinarian

SSI, surgical site infection

### Hospital protocols for surgical asepsis and use of antimicrobials in clean orthopaedic and neurosurgeries

The fur was clipped, and the surgical site was first aseptically scrubbed with disposable swabs using 4% chlorhexidine gluconate (Hibiscrub, BCM Ltd., United Kingdom) then wiped with 70% (v/v) ethanol (Erisan Dermades, KiiltoClean Oy, Finland) in a designated preparation room. The dog was then transported to the operating room where the surgical site was again wiped with 70% ethanol. The surgical site was covered with disposable sterile drapes and an adhesive film (Opsite, Smith&Nephew, United Kingdom).

The surgery rooms were reserved for clean surgeries only, and a maximum of seven persons were allowed in the room. All personnel and observers in the operating room wore a surgical mask and cap. All members of the surgical team followed the same aseptic preparation of hands and arms before entering the operating room. The surgical hand preparation was performed according to the WHO guidelines [21]. The primary surgeon and assistant(s) wore a sterile surgical gown and gloves. Double gloving was used in all orthopaedic surgeries. The gloves were changed during surgery if any disruption in aseptic technique was suspected.

The anaesthetic protocol and pain medication were planned individually for each patient by an anaesthesiologist. To comply with the hospital protocol for prophylactic antibiotic administration, the antimicrobial was given intravenously 30–60 min before the estimated time of incision. The first-choice antimicrobial was cefazolin at a dose of 22 mg/kg body weight (BW), and the dose was repeated at 90 min intervals until the end of surgery. After surgery, the incision was covered with a sterile bandage. After extubation, the dog was transported to the recovery room, where it was closely monitored for post-anaesthetic complications and pain until fully recovered. Postoperative antimicrobials were not routinely administered. The dog was either discharged or transferred to the intensive care unit for postoperative monitoring.

### Data and statistical analysis

Data were entered into an Epi-Info database (v. 7.2, CDC). Data validation included visual inspection and checking certain data (date and time variables; antimicrobial use for the treatment of SSI and the name of the drug) for logical consistency by programmed SAS scripts. After correcting discrepancies (three errors in time of hospitalization and one error in the date of surgery), a randomly selected sample of 20 patients was used for independent quality control by comparing the sample with the data in the electronic patient records. The accepted error rate was 0% for critical and < 0.5% for other data. The critical variables were the carriage of MDR bacteria, presence of an implant, antimicrobial administration, systemic disease, presence of SSI, the result of a bacterial culture for SSI, treatment of SSI, and consequences of SSI.

Descriptive statistics of the entire data were presented as frequency and percentage distributions for categorical variables; for continuous variables, data were presented as mean, standard deviation (SD), and range values. To assess potential risk factors associated with SSI, univariable and multivariable logistic regression models were used. The dependent variable was the presence or absence of SSI. Other variables served as independent variables. Univariable and multivariable logistic regression analyses were used to examine the probability of infection using Firth’s bias adjustment method to take into account the rareness of the events [22]. When dogs had several interventions on different dates, each intervention was recorded as a separate surgery and the interventions were addressed individually in statistical analysis. Factors that were identified as meaningful in the univariable model (P < 0.1) were inserted into a multivariable logistic regression model, where the main effects of these factors were all simultaneously included as fixed effects; no interactions between the factors were studied due to the rareness of outcome events. Main conclusions were drawn from the multivariable model. For all analyses, a P-value of < 0.05 was considered significant. All statistical analyses were performed using SAS^®^ System for Windows (v. 9.4, SAS Institute Inc., Cary, NC, USA).

## Results

Data were collected for 406 dogs, of which 202 (49.8%) were females and 204 (50.2%) were males. A total of 101 different breeds were included; mixed breed dogs were most common (50), followed by Dachshunds (23) and Labrador retrievers (13). The mean ± SD age was 7.6 ± 3.5 (range 2.0–18.0) years and BW was 16.5 ± 14.0 (range 1.0–96.0) kg. All dogs were classified as ASA class 2. Screening for MDR bacteria had been preoperatively performed on 26 dogs (i.e. classified as risk patients), of which six were found to be carriers of methicillin-resistant *Staphylococcus pseudintermedius* (MRSP). Surgical procedures performed are presented in Table 2.

**Table 2:** Use of antimicrobials in different surgical procedures, included to evaluate the compliance with existing national and in-house guidelines

Surgical procedure	No. of dogs	Dogs receiving antimicrobials			Dogs not receiving antimicrobials	Dogs with missing data regarding the time of antimicrobial administration	Dogs receiving antimicrobial prophylaxis between 30 and 60 min before incision
		Preop^a^	Prophylaxis^b^	Postop^c^			
All	406	5	377	4	29	20	215/357^d^ (60.2%)
Fracture stabilization	108	2	102	2	6	6	61/96^d^ (63.5%)
Cranial cruciate ligament rupture repair	92	1	80	1	12	4	38/76^d^ (50.0%)
*TPLO*^*e*^	71	1	60	1	11	3	24/57^d^ (42.1%)
*Extracapsular repair*	21	–	20	–	1	1	14/19^d^ (73.7%)
Patellar luxation repair	57	2	52	–	5	1	32/51^d^ (62.7%)
Hemilaminectomy and laminectomy	54	–	54	1	–	3	30/51^d^ (58.8%)
Arthroscopy	39	–	36	–	3	4	21/32^d^ (65.6%)
Ulnar ostectomy	18	–	16	–	2	1	10/15^d^ (66.7%)
Arthrodesis	14	–	14	–	–	1	7/13^d^ (53.8%)
Femoral head and neck excision	13	–	12	–	1	–	8/12^d^ (66.7%)
Arthrotomy	6	–	6	–	–	–	4/6^d^ (66.7%)
Coxofemoral luxation repair	5	–	5	–	–	–	4/5^d^ (80%)

^a^Preop, preoperatively: before admission to surgery

^b^Parenteral administration of antimicrobials in the immediate proximity to surgery. This group also included dogs with preoperative and postoperative antimicrobial administration

^c^Postop, postoperatively: after surgery or discharge

^d^Number of dogs with information regarding the time from antimicrobial administration to skin incision

^e^Tibial plateau levelling osteotomy

### Antimicrobial administration

Altogether, 377/406 dogs (92.9%) received prophylactic antimicrobials (Table 2). Five of the 377 dogs also received antimicrobials during the week preceding surgery for a disease unrelated to the surgical procedure. Further, four dogs (1.1%) received surgery-related prophylactic antimicrobials as well as postoperative antimicrobials due to revision fracture stabilization surgery (2), recurrent urinary tract infections (1), and suspected aspiration pneumonia (1). Nearly all of the dogs that received surgical prophylaxis (370/377, 98.1%) were administered cefazolin. Dogs known to be carriers of MRSP (n = 6) did not receive cefazolin. Instead, they received amikacin (3), clindamycin (1), enrofloxacin (1), or trimethoprim/sulfamethoxazole (1) according to the susceptibility profile of the MRSP bacterial strain. In addition, one dog received ampicillin.

The time from prophylactic antimicrobial administration to skin incision was available for 357/377 dogs (94.7%) (Table 2). Altogether, 215/357 dogs (60.2%) received antimicrobial prophylaxis 30–60 min before incision, according to the hospital protocol; 87 dogs (24.4%) received it > 60 min before incision, 41 dogs (11.5%) received it < 30 min before incision, and 14 dogs (3.9%) received it after the surgical incision. Antimicrobial prophylaxis was repeated according to hospital protocol during the surgery (i.e. at 90 min intervals) in 306/377 dogs (81.2%).

### Intraoperative variables

Information regarding the duration of anaesthesia and surgery was available for 320/406 dogs (78.8%) and 382/406 dogs (94.1%), respectively. Transient hypotension was reported in 126/406 dogs (31.0%). Further data on intraoperative variables are presented in Tables 3 and 4.

**Table 3:** Intraoperative variables describing the representativeness of the data

Variable	All dogs (406)	Dogs with follow-up (363)
	Mean ± SD^a^ (range)	Mean ± SD (range)
Duration of anaesthesia (min)	245.7 ± 75.1 (100–495)	248.7 ± 76.4 (100–495)
Duration of surgery (min)	126.9 ± 54.3 (20–345)	127.4 ± 53.8 (20–345)
Lowest MAP^b^ (if < 60 mmHg) during surgery (mmHg)	49.5 ± 5.2 (35–55)	49.5 ± 5.0 (35–55)
Duration of hypotension (MAP < 60 mmHg) (min)	26.4 ± 22.0 (5–125)	26.0 ± 21.5 (5–125)
Lowest body temperature during surgery (°C)	36.1 ± 0.9 (33.2–39.7)	36.1 ± 0.9 (33.2–39.7)
	Yes (%)	Yes (%)
Antimicrobial administered	377 (92.9)	337 (92.8)
Implant placed	279 (68.7)	251 (69.1)
Propofol administered	349 (86.0)	311 (85.7)

^a^SD, standard deviation

^b^MAP, mean arterial pressure

**Table 4:**
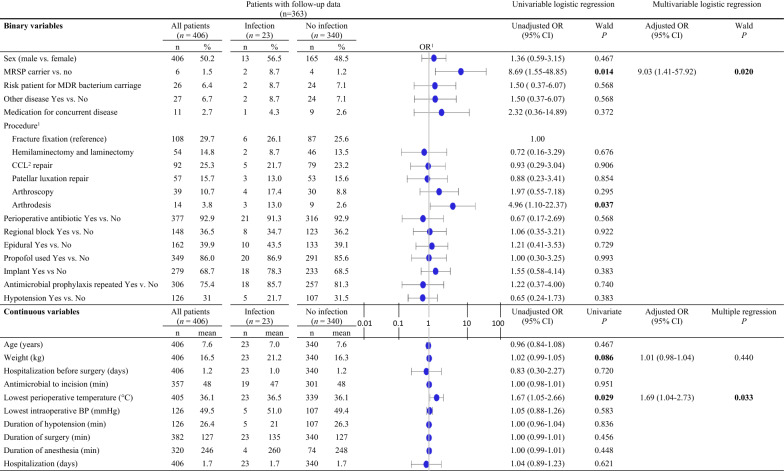
Univariable and multivariable logistic regression results for the risk factors associated with surgical site infection

^1^Only procedures with at least one infection were included

^2^Cranial cruciate ligament

### Occurrence and treatment of SSI

Only dogs with follow-up information (363/406, 89.4%) were included in further analysis of SSI occurrence. Mean follow-up time was 464 days (range 3–2600) (Table 5). SSI was diagnosed in 23/363 dogs (6.3%), of which 10 (43.5%) had a superficial and 13 (56.5%) a deep infection. Mean time of SSI diagnosis was 68 days (median 15; range 4–522). Ten cases of SSI were seen within the first 2 weeks after surgery. In orthopaedic surgeries, the SSI rate was 6.7% (21/315), with the highest frequencies in arthrodesis (3/12, 25.0%) and arthroscopy (4/34, 11.8%), whereas in TPLO the SSI rate was 7.7% (5/65), in fracture stabilization 6.5% (6/93), and in neurosurgeries 4.2% (2/48). Due to SSI, 11 dogs underwent revision surgery, and the implant was removed from eight of these dogs. One dog was euthanized due to SSI at the owner’s request. Remarkably, none of the 26/363 dogs (7.2%) that did not receive any antimicrobials got an SSI. Bacterial culture was taken in 15/23 (65.2%) SSI cases; all of these cultures were positive. The most commonly cultured bacterium was *S. pseudintermedius* (n = 9), one of which was MRSP. Single cases of *Escherichia coli*, Group B streptococci, *S. aureus*, *Pasteurella* sp., *Corynebacterium* sp., and *Enterococcus faecalis* were found. Two of the dogs diagnosed with SSI were carriers of MRSP; in these two dogs, the SSI was caused by MRSP and susceptible *S. pseudintermedius*, respectively.

**Table 5:** Data on the follow-up time, SSI rates, time of SSI diagnosis, and antimicrobial administration protocol in different groups of procedures

Surgical procedure		Dogs with follow-up information (n)	Follow-up time mean (range) (days)	SSI^a^ diagnosed n (%)	Time of SSI diagnosis mean (range) (days)	Antimicrobial administration	
						Yes (n)	No (n)
All		363	464 (3–2600)	23 (6.3)	68 (4–522)	337	26
Fracture stabilization		93	476 (3–2600)	6 (6.5)	30 (4–82)	87	6
Cranial cruciate ligament repair	TPLO^b^	65	588 (12–1859)	5 (7.7)	235 (15–522)	56	9
	Extracapsular stabilization	19	395 (11–2594)	0	–	18	1
Patellar luxation repair		56	425 (11–2147)	3 (5.4)	13 (12–15)	51	5
Hemilaminectomy and laminectomy		48	393 (5–2051)	2 (4.2)	7 (7)	48	0
Arthroscopy		34	515 (11–2201)	4 (11.8)	13 (11–16)	31	3
Ulnar ostectomy		17	336 (30–963)	0	–	16	1
Arthrodesis		12	288 (40–1031)	3 (25)	32 (5–71)	12	0
Femoral head and neck excision		10	562 (14–2041)	0	–	9	1
Arthrotomy		6	592 (58–1594)	0	–	6	0
Coxofemoral luxation repair		3	219 (12–397)	0	–	3	0

^a^SSI, surgical site infection

^b^TPLO, tibial plateau levelling osteotomy

Altogether, 16/23 dogs with SSI were treated with systemic antimicrobials: cephalexin (n = 7), amoxicillin/clavulanic acid (n = 4), clindamycin (n = 3), trimethoprim/sulfamethoxazole (n = 1), and amoxicillin and clindamycin (n = 1). Seven dogs were treated with only topical agents.

### Risk factors for SSIs

In the risk factor analysis, omission of antimicrobial administration did not increase the risk for SSI (P = 0.56; OR 1.7; CI_95%_ 0.4–5.0). However, MRSP carriage (P = 0.02; OR 9.0; CI_95%_ 1.4–57.9) and perioperatively measured higher body temperature (P = 0.03; OR 1.69; CI_95%_ 1.0–2.7; mean difference + 0.4 °C compared to dogs without SSI) were shown to be risk factors for SSI (Table 4). In the univariate analysis, pairwise comparisons of arthrodesis and fracture fixation indicated that arthrodesis could increase the risk of SSI compared to fracture fixation, as the nominal P-value was statistically significant (P = 0.04; OR 4.96; CI_95%_ 1.1–22.4). However, the overall test for differences between procedures in the risk of SSI was statistically insignificant (P = 0.21).

## Discussion

The overall SSI rate in our study was 6.3%, which can be considered satisfactory compared with previous studies. Of all patients, 92.9% received antimicrobial prophylaxis. This reflects good compliance with in-house and national antimicrobial protocols, which underline that no postoperative antimicrobials are recommended in clean orthopaedic or neurosurgeries [9].

According to our findings, prophylactic antimicrobial administration is sufficient to maintain SSI rates at an acceptable level in clean orthopaedic and neurosurgeries. Since only four dogs received postoperative antimicrobials, we could not investigate the effect of postoperative antimicrobial administration on SSI rate. Nevertheless, our data indicate that even complete omission of antimicrobials was not associated with increased risk for SSI. It is important to note that since very few patients (7.2%) did not receive any antimicrobials, the risk estimate for performing the surgery without antimicrobials is uncertain. Strict aseptic practice, proper surgical technique, balanced anaesthesia, and careful SSI surveillance are key issues in SSI prevention. Although single doses of antimicrobial prophylaxis for an individual lead to minimal antimicrobial exposure, the cumulative amount of drugs used in animals undergoing surgery is significant, and may impact the resistance on a population level. Therefore, more research on the impact of diminishing antimicrobials in veterinary surgeries is warranted.

Compliance with antimicrobial protocols regarding the choice of prophylactic antimicrobial was excellent, as cefazolin was administered for nearly all dogs receiving antimicrobial prophylaxis. Cefazolin is the recommended antimicrobial in orthopaedic surgery due to its rapid equilibrium between plasma and surgical wound fluid, favourable antimicrobial spectrum, sustained tissue concentrations, low toxicity, and low cost [23, 24]. In our study, 71.7% of the dogs received the first antimicrobial within 60 min before surgical incision. This is in concordance with a previous study by Weese and Halling [25], in which 70.5% of the patients received antimicrobials within 60 min before surgery. The mean time between the administration of the first antimicrobial dose and the surgical incision was 48 min, which is within the recommended time frame of 30–60 min [23, 26]. Although timing of the prophylaxis was suboptimal in one-third of the cases, the timing of antimicrobial prophylaxis was not shown to be a risk factor for SSI in our study.

One of the encouraging findings in our study was that the SSI rate after TPLO surgeries was 7.7% without the use of postoperative antimicrobials. In previous studies, the SSI rate without administration of postoperative antimicrobials has been reported to be 19.7% [15], 17.0% [17], 10.7%, [10] and 2.5% [13]. Compared with these studies, the SSI rate of TPLO surgeries in our study is fairly low. Based on the literature, the risk of SSI after TPLO surgeries appears to be higher than that of other clean orthopaedic procedures. Reported SSI rates range from 2.9% to 25.9% [10, 12, 13, 15, 17, 27, 28]; postoperative antimicrobial administration has been shown to be a protective factor against SSI in most of these studies [10–13, 15, 28]. However, due to increasing concern regarding antimicrobial resistance, it is crucial to pay attention to a rigorous aseptic technique and other factors that affect the SSI rate in order to minimize the importance of antimicrobial use. In TPLO surgeries, the elevated SSI rate has been attributed to the use of non-locking plates [15], aggressive periosteal dissection, reduced soft tissue coverage of the proximal tibia, thermal bone necrosis during osteotomy, and presence of an implant [27, 29]. In addition, TPLO without using a jig predisposes patients to inaccurate osteotomy angulation, fibular fracture, and fixation failure [30, 31].

Displeasingly high SSI rates were found in our study after arthroscopies (4/34; 11.8%) and arthrodeses (3/12; 25%), although the total number of these types of surgeries was low. The SSI rate after joint arthrodeses in previous reports has ranged from 2.6% to 18.2% [32–34], but the use of antimicrobials was not reported in these studies. Our data did not reveal reported risk factors such as duration of anaesthesia, surgical time or intraoperative hypotension that would explain the high SSI rate in our data [19, 35]. Further, due to the retrospective nature of the study, information not mentioned in the patient records could not be assessed. Therefore, no specific reason for the high SSI rate in these types of surgeries could be identified. In attempt to shed light on the high rate of SSIs in arthroscopies in this study, we reviewed more recent data on an additional 28 arthroscopies with follow-up information from 2017 to 2018. This review revealed that none of these arthroscopies resulted in a SSI. In any case, this is a subject warranting further investigations.

Earlier reports on SSI rate in neurosurgeries are scarce. In the study by Dyall et al. [18] the SSI rate was only 0.6% without any use of antimicrobials, which is exceptionally low compared with other clean surgeries. In human medicine, the reported SSI rate ranges from 0.7 to 12% [36]. In our study, the SSI rate in neurosurgeries was 4.2%, which can be considered satisfactory.

In our study, carriage of MRSP was shown to be a risk factor for SSI in the multivariate analysis, although the number of patients was low, as demonstrated by the wide confidence interval. Carriage of MRSP has also been shown to be a risk factor in a study by Nazarali et al. [13]. Patients carrying MRSP often have a history of numerous or prolonged antimicrobial therapies or underlying skin or ear problems, as MRSP is frequently isolated from the skin of dogs with active or recent skin disease [37, 38]. Further, beta-lactam antimicrobials, e.g. cefazolin, are ineffective against MRSP [39]. In our study, screening for MDR bacteria had been performed on the 26 dogs that were classified as risk patients. Six of these were found to be MRSP carriers. Risk patients (those with a history of recurrent ear or skin infection, prolonged or numerous hospital visits or antimicrobial treatments, as well as patients with SSI or suppurative wound infection or contact with an animal carrying MDR bacteria) are screened in our hospital for MDR bacteria such as MRSP on admission. Previous data show that the MRSP carriage rate is higher (9%) in these patients than in low-risk patients i.e. the general Finnish dog population (3%) [37, 40, 41]. There is no reason to expect that the MRSP carriage rate would be higher in the non-screened patients in our study than in the general population. However, this assumption is prone to error and should be further investigated in future prospective studies. Active surveillance of MRSP has been carried out in our hospital since 2010.

Surprisingly, lower body temperature was shown to be a protective factor for SSI in multivariable analysis. This is contradictory to previous reports, and without further evidence we are inclined to believe that this is an incidental finding. Perioperative hypothermia may lower resistance to infection by inhibition of leukocyte migration, phagocytosis, and decreased cytokine synthesis, predisposing to SSI [42]. However, a retrospective study on 777 dogs and cats found no significant difference in the analysis of temperature data between animals with infected and uninfected wounds [43].

The main limitation of the study was the retrospective design, and the inclusion of data from several different procedures. However, the latter was considered necessary to reach adequate statistical power for the risk factor analysis. Due to the retrospective design, missing data in some variables may introduce bias to our results. Data regarding the duration of anaesthesia and surgery were missing in 21.2% and 5.9% of the dogs, respectively; for 10.6% of dogs, there was no follow-up information. The preoperative variables of the dogs with no follow-up information did not differ from the rest of the data, so we could assume that the SSI rate in this population does not differ significantly from that of the dogs with follow-up information.

This study identified several hospital practices that should be improved. The practice of performing bacterial culture in cases with suspected SSI should be reinforced, as it was performed in only 65.2% of the dogs with SSI. Anaesthesia and surgical records should be kept more meticulously, and the timing of antimicrobial administration should be more accurate. Surgical safety checklists have been shown to reduce the risk of SSIs, [44] and the use of a checklist has now been implemented in our hospital.

## Conclusions

With the Nordic antimicrobial policy of using only prophylactic antimicrobial administration in canine orthopaedic and neurosurgeries, the overall SSI rate was satisfactory and comparable to previously published data. However, as there was variation in the SSI rate between different procedures, it should be further evaluated if certain procedures, such as arthrodesis, would benefit from postoperative antimicrobial administration. The compliance with in-house and national protocols was excellent regarding both the adherence to using prophylaxis only in these procedures and the choice of prophylactic antimicrobial, but there was room for improvement in the timing of prophylaxis. Carriage of MRSP was shown to be a significant risk factor for SSI. More detailed and prospective studies are warranted to investigate the need for prophylaxis in veterinary clean orthopaedic surgeries.

## Supplementary information

**Additional file 1:** Risk based classification of patients at the Small Animal Hospital of Helsinki University.

## Data Availability

The datasets used and/or analysed during the current study are available from the corresponding author on reasonable request.
