# Supplementary material for: Antimicrobial prophylaxis is considered sufficient to preserve an acceptable surgical site infection rate in clean orthopaedic and neurosurgeries in dogs
Source: Acta Vet Scand. 2020 Sep 17;62:53. doi: 10.1186/s13028-020-00545-z (PMC7495856; doi:10.1186/s13028-020-00545-z)
Supplement: Supplementary file 1 — Additional file 1: Risk based classification of patients at the Small Animal Hospital of Helsinki University. [file 13028_2020_545_MOESM1_ESM.pdf]

Additional file 1. Risk based classification of patients at the Small Animal Hospital of Helsinki University

**Classification**	**Criteria**
High-risk patients	MRSP[1]-positive<br>Has been hospitalized > 24 hours and has signs of a hospital acquired infection
Medium-risk patients	Has a history of recurrent ear or skin infection<br>Has a history of prolonged or numerous hospital visits or visits at other veterinary clinics<br>Has a history of prolonged or numerous antimicrobial treatments<br>Has been exposed to a patient with MRSP<br>Has had surgery elsewhere and has a surgical site infection<br>Has a suppurative wound infection
Low-risk patients	All other patients

[1] Methicillin-resistant *Staphylococcus pseudintermedius*
